# Papillomavirus vaccine: a revolution in the fight against cervical cancer in the Democratic Republic of Congo

**DOI:** 10.1097/MS9.0000000000003492

**Published:** 2025-06-25

**Authors:** Christian Tague, Hermann Yokolo, Dujardin Makeda, Joshua Ekouo, Elie Kihanduka, Aymar Akilimali

**Affiliations:** Department of research, Medical Research Circle (MedReC), Goma, DR Congo

Cervical cancer is a major public health problem, particularly in the Democratic Republic of Congo (DRC) and other developing countries. According to the World Health Organization (WHO), the disease is the fourth leading cause of cancer in women worldwide, but it is particularly deadly in low- and middle-income countries. In the DRC, where access to preventive care and screening is limited, cervical cancer is one of the leading causes of female cancer mortality^[1]^. In many developed countries, routine vaccination of girls and, more recently, boys, has significantly reduced HPV infections and associated precancerous lesions. In the DRC, the introduction of the vaccine represents a major opportunity to reduce the burden of cervical cancer and improve women’s long-term health^2^ The objective of this article is to examine the impact of the HPV vaccine in preventing cervical cancer and to discuss the public health implications of this advance.

## HPV infection and vaccination in the Democratic Republic of Congo

In the DRC, HPV infection is a major concern, especially due to the high prevalence of oncogenic types such as HPV 16 and 18, which are responsible for the majority of cervical cancer cases globally. Despite the burden, HPV genotyping and systematic screening are rarely performed, particularly in rural and underserved areas. Cytological screening programs are limited, with most diagnoses made at advanced stages of disease, contributing to high mortality rates^[1,3,4]^.

The government of the DRC, with support from international partners such as GAVI and the WHO, has initiated steps toward integrating HPV vaccination into the national immunization program. In 2023, the DRC launched its first nationwide HPV vaccination campaign, targeting girls aged 9 to 14. The quadrivalent vaccine (Gardasil), which protects against HPV types 6, 11, 16, and 18, is the most commonly used in the country^[5-7]^.

However, several challenges hinder effective implementation. These include logistical difficulties in reaching remote populations, limited cold chain capacity, and a shortage of trained healthcare workers. Moreover, sociocultural resistance and misinformation about the vaccine persist. Surveys conducted in Kinshasa and other provinces have shown varying levels of acceptance, with concerns about vaccine safety, misconceptions about infertility, and reluctance to discuss sexual health with young girls^[3-7]^.

Despite these barriers, initial vaccination campaigns have been moderately successful in urban settings, where school-based strategies were adopted. Community engagement and awareness campaigns, often involving religious and community leaders, are essential to improve coverage. The national goal is to reach at least 80% of eligible girls by 2030, in alignment with the WHO global strategy to eliminate cervical cancer^[1,3]^.

Human papillomavirus (HPV) is a group of viruses belonging to the Papillomaviridae family. There are more than 200 types of HPV, approximately 40 of which affect the anogenital region and are transmitted through sexual contact. HPV is primarily transmitted through skin-mucosal contact, particularly during sexual intercourse (vaginal, anal, or oral)^[1]^. It is extremely contagious: a single exposure can be enough to cause infection. According to the WHO, cervical cancer is a major public health problem, particularly in low- and middle-income countries. In 2022, it caused approximately 660 000 new cases and 350 000 deaths worldwide, accounting for more than 90% of HPV-related cancers in women. Sub-Saharan Africa is the most affected region (24% prevalence). Women living with HIV, immunocompromised people, and people without access to testing are the most vulnerable. Inequality in access to vaccination and care exacerbates the situation, leading to high and preventable mortality^[3]^.

Vaccination against the HPV has revolutionized cervical cancer prevention by significantly reducing infections and the incidence of the disease. A U.S. study found that between 1999 and 2017, HPV vaccination led to a significant decrease in cervical cancer cases among young women. Squamous cell carcinoma rates fell by 12.7% per year among women aged 15 to 20, by 5.5% among those aged 21 to 24, and by 2.3% among those aged 25 to 29. The most significant decline was observed between 2010 and 2017 among 15- to 20-year-olds, with a reduction of 22.5% per year. Furthermore, adenocarcinoma rates have also declined, reaching an annual decrease of 9.4% between 2006 and 2017 among younger people. This reduction in cases also has a major economic impact, limiting the costs associated with cervical cancer care and treatment. In the United States, widespread vaccination could save several billion dollars in medical costs. In developing countries, such as those in sub-Saharan Africa where access to care is limited, the introduction of the vaccine could prevent thousands of deaths each year and ease the burden on health infrastructure^[1,3]^.

Despite the proven efficacy of the HPV vaccine, its accessibility and vaccination coverage remain uneven across the world. In developed countries, where school vaccination programs and well-structured public health policies exist, vaccination coverage often reaches high levels. For example, in Australia and the United Kingdom, more than 80% of adolescent girls have received at least one dose of the vaccine, leading to a significant decrease in HPV infections and precancerous lesions^[5]^.

Receptivity among the Congolese population to HPV vaccination remains low, particularly in rural areas and among conservative religious communities. A qualitative study conducted in Kinshasa in 2023 revealed that less than 40% of parents understood the preventive role of the cervical cancer vaccine. Fear of side effects, a lack of clear information, and weak engagement of community leaders were identified as major factors driving reluctance. Targeted and culturally appropriate awareness campaigns are needed to increase acceptability^[1,3-6]^.

In contrast, in developing countries, access to the vaccine is limited by several factors, including its high cost, the lack of adequate health infrastructure, and logistical distribution challenges. In sub-Saharan Africa, vaccination coverage remains low, often below 20%, due to lack of funding for vaccination campaigns and challenges related to vaccine storage and administration in rural areas. The WHO global initiative to eliminate cervical cancer by 2030 encourages the establishment of vaccination programs, but their rollout remains a challenge^[6]^.

The national program aims to achieve vaccination coverage of at least 90% among girls aged 9 to 14 by 2030, in line with the WHO global strategy to eliminate cervical cancer. In the medium term, the goal is to make the vaccine available in all provinces and to combine it with a structured screening program using HPV testing or cervical smears, particularly for women over 30. The establishment of vaccination registries and an epidemiological monitoring system is also planned^[5-7]^.

## Long-term vaccine effectiveness and feedback

Longitudinal studies conducted in countries such as Rwanda, Australia, and the United Kingdom have demonstrated a significant reduction in the incidence of precancerous lesions and cervical cancer within 10 to 15 years following the introduction of the HPV vaccine. These data confirm the long-term efficacy of the vaccine, particularly when administered before sexual exposure. Although the DRC does not yet have local post-vaccination data, these international experiences strengthen the rationale for rapid and widespread implementation of vaccination^[1,3-7]^.

Beyond logistical and financial challenges, sociocultural barriers also hamper HPV vaccine uptake. In many regions, vaccine hesitancy is based on misconceptions, including the idea that vaccinating girls against a sexually transmitted infection would encourage early sexual activity. This perception is particularly prevalent in some conservative communities, where discussing sexual health remains taboo^[1,5]^.

Initially targeted at young girls due to the direct association between HPV and cervical cancer, vaccination is gradually being extended to boys in several countries. HPV is also responsible for other types of cancer, including anal, throat, and penile cancer, which also affect men. Extending vaccination to boys has several advantages. It not only directly protects men against HPV-related diseases but also strengthens herd immunity by reducing transmission of the virus. However, in many developing countries, where resources are limited, the priority remains to vaccinate young girls, considered the most at-risk population. Nevertheless, for sustainable HPV eradication, vaccinating boys must be considered as a complementary long-term lever^[1^-^7]^.

Future research aims to develop vaccines that cover a wider range of HPV types and explore therapeutic vaccines to treat existing infections. In the long term, high global vaccination coverage could significantly reduce mortality from cervical cancer and other HPV-associated cancers. However, challenges remain, particularly in low-resource settings, where access to the vaccine remains limited. International initiatives, such as those supported by WHO and GAVI, seek to overcome these barriers by making the vaccine more accessible and affordable. To maximize the overall impact, it is crucial to improve local infrastructure and integrate the vaccine into global public health programs^[1,3,8]^.

## Outlook and Recommendations for the DRC

To maximize the impact of HPV vaccination in the DRC, several avenues can be considered. It is essential to integrate vaccination into the national school curriculum, train community health personnel, and involve religious and traditional leaders in awareness-raising. Continued technical and financial support from international partners is also necessary to ensure vaccine availability and conservation. In the long term, the program’s success will depend on a multisectoral approach integrating vaccination, screening, early treatment of precancerous lesions, and rigorous epidemiological monitoring.

In conclusion, the HPV vaccine has proven effective in reducing HPV infections, precancerous lesions, and cervical cancers, with particularly notable results in countries with high vaccination coverage. It has also shown potential in preventing other HPV-related cancers, such as anal and throat cancers. However, challenges remain, particularly in low-resource countries where access to the vaccine remains limited. It is essential to strengthen vaccination programs, overcome barriers to access, and promote vaccination in underserved populations. With widespread global vaccination coverage, it is possible to eliminate a large proportion of HPV-related cancers and significantly reduce associated mortality. Vaccination plays a crucial role in the fight against these cancers, and its widespread deployment represents a key objective for the future of public health.

## Data Availability

Not applicable.
